# Transient Intraocular Pressure Fluctuations After Intravitreal Bevacizumab Injection in Proliferative Diabetic Retinopathy Patients: A Prospective Study

**DOI:** 10.7759/cureus.45371

**Published:** 2023-09-16

**Authors:** Faizan Luqman, Hafsa Bibi, Marwa Mukhtar, Fahad Zafar, Hafsa Ahmed, Muhammad A Khizer, Nazli Gul

**Affiliations:** 1 Ophthalmology, Khyber Medical College, Peshawar, PAK; 2 Ophthalmology, Medical Teaching Institution (MTI) Khyber Teaching Hospital, Peshawar, PAK; 3 Ophthalmology, Medical Teaching Institute (MTI) Khyber Teaching Hospital, Peshawar, PAK; 4 Ophthalmology, Medical Teaching Institute (MTI) Ayub Teaching Hospital, Abbottabad, PAK; 5 Ophthalmology, National University of Medical Sciences, Rawalpindi, PAK; 6 Ophthalmology, Armed Forces Institute of Ophthalmology, Rawalpindi, PAK

**Keywords:** transient iop fluctuations, intraocular pressure changes, immediate iop fluctuations, post-ivb injection iop, short-term iop change, iop, ivb, intravitreal bevacizumab injection, pdr, proliferative diabetic retinopathy

## Abstract

Introduction: The clinical use of intravitreal bevacizumab (IVB), a recombinant humanized monoclonal antibody that functions as an anti-vascular endothelial growth factor (anti-VEGF), has recently increased in patients with retinal ischemic diseases such as proliferative diabetic retinopathy (PDR). The short-term and long-term complications associated with this procedure have not been well established. We aimed to study the possible short-term complication of intraocular pressure (IOP) fluctuations shortly after IVB injection in patients with PDR.

Materials and methods: A prospective case series of diabetic patients with PDR who underwent IVB injection was performed in the Department of Ophthalmology, Medical Teaching Institution, Khyber Teaching Hospital, Peshawar, Pakistan, from November 1, 2020, to May 1, 2021. The total number of PDR patients of both sexes included in the study was 101. A slit lamp examination was performed, and IOP readings were recorded before and 30 min after IVB injection using Goldmann applanation tonometry (GAT). IBM Statistical Package for the Social Sciences version 22 for Windows was used to analyze the data. Safety of the procedure, defined as IOP *≤*20 mmHg 30 min after IVB injection, was determined and stratified according to sex, age, duration of diabetes, and baseline IOP. A post-stratification chi-square test was applied, and a p-value <0.05 was taken as statistically significant.

Results: In this study, 60.4% of the participants were male and 39.6% were female. The age of the patients ranged from 30 to 75 years, with a mean age of 55.66±6.37 years. The mean duration of diabetes among the participants was 7.73±2.94 years and the mean baseline IOP was 15.40±1.77 mmHg. Safety (IOP *≤*20 mmHg 30 min after IVB injection) was observed in 90.1% of the patients.

Conclusion: IVB injections are safe for use in patients with PDR in terms of immediate IOP changes. However, patients with higher baseline IOP (>15 mmHg) are more likely to develop increased IOP post-procedure and prophylaxis may be prudent in such cases.

## Introduction

Diabetes mellitus (DM) is a major health concern worldwide and is spreading like wildfire. Approximately 135 million people had DM in 1995, which was estimated to rise to 300 million by the year 2025 [1]. However, the number of diabetics reached 336 million in 2011. This sudden and unexpected increase changed the prediction to 552 million cases of DM by 2030. Chronic high blood glucose levels cause microvascular and macrovascular damage in the body; one of the most common microvascular diseases is diabetic retinopathy (DR) [2]. The severe form of DR is known as proliferative diabetic retinopathy (PDR), which involves dysfunctional and disorganized retinal vascularization. DR is the major cause of visual impairment among the working-age population. According to meta-analyses of population-based studies, the number of individuals with vision problems and complete vision loss due to DR is increasing rapidly [3,4].

Primary open-angle glaucoma and DM are commonly observed together according to the epidemiological data [5]. Patients with uncontrolled DM are more likely to have significantly higher intraocular pressure (IOP) than those with well-controlled diabetes [6]. The exact pathophysiology is not well understood, but some experimental studies point toward the excess formation of extracellular matrix in the trabecular meshwork, which eventually blocks the drainage of aqueous humor and causes high IOP [7].

Currently, ophthalmologists favor using intravitreal anti-vascular endothelial growth factor (anti-VEGF) to treat various ischemic retinal diseases such as DR and retinal vein occlusions, unless there is a contraindication or patient refusal [8]. Bevacizumab (Avastin®, Genentech Inc., CA, USA) is a humanized monoclonal antibody that antagonizes all isoforms of VEGF-A by binding to it and preventing its attachment to VEGF receptors [9].

The eyeball is a closed cavity, and injecting any extra volume of fluid into the vitreous cavity is believed to increase the IOP. A study published in 2014 showed frequent IOP spikes after intravitreal bevacizumab (IVB) injection for PDR, but the change in IOP was clinically insignificant, and no anti-glaucoma medication was recommended pre- or post-procedure, except in the situation where IOP remained high (>25 mmHg) after two hours [10]. As there has been an exponential increase in the number of patients with uncontrolled DM, indicating an increase in the number of patients with PDR, IVB injections are becoming more common. Therefore, checking the safety of this procedure, particularly sudden spikes in IOP, is vital.

In this study, we focused on immediate IOP changes in patients with PDR after IVB injections. We also tried to find an association between post-IVB injection IOP changes and other variables, such as sex, age, duration of diabetes, and baseline IOP, which, to our knowledge, has never been done before.

## Materials and methods

A prospective case series of diabetic patients with PDR who underwent IVB injection was performed in the Department of Ophthalmology, Medical Teaching Institution, Khyber Teaching Hospital, Peshawar, Pakistan, from November 1, 2020, to May 1, 2021.

The study was commenced after obtaining ethical approval from the Ethics Committee of Khyber Teaching Hospital endorsed by the College of Physicians and Surgeons of Pakistan (Ref. No. CPSP/REU/OPL-2019-020-2037). A total of 101 patients of both sexes with PDR were included in this study. The WHO sample size calculator was used with a 95% confidence interval, 8% margin of error, and the expected prevalence of normal IOP of 78.8% after 30 min of IVB injection for PDR to calculate the sample size [10]. A non-probability consecutive sampling technique was used. Diabetic patients with PDR who underwent IVB injections with a baseline IOP <20 mmHg on Goldmann applanation tonometry (GAT) between the ages of 30 and 75 years were included. Patients with a history of any ocular procedure (including routine cataract surgery), intravitreal steroid injections within the previous three months, and those who refused to give informed consent were excluded from our study.

Demographic information of the patients, including sex, age, and duration of diabetes, along with written informed consent, were obtained, ensuring the confidentiality and safety of the participating patients in this study. After measuring the baseline IOP of the participating patient, intravitreal injection was administered using a standard technique [11]. The patient was placed in the supine position, and a solution of 5% povidone-iodine was applied to the eyelids and eyelashes, instilled in the conjunctival sac for 3-5 min, and washed with normal saline to sterilize the area. Draping was performed and a wire lid speculum was applied for lid control. A pre-filled 0.05 mL IVB injection (1.25 mg) was administered with a 30-gauge needle through the pars plana 3.5-4.0 mm posterior to the limbus using a sterile ophthalmic caliper. The needle was inserted into the globe in a step-like manner by pulling the conjunctiva away using a sterile cotton swab, and the drug was injected into the vitreous cavity. The injection site was pressed using the same sterile cotton swab to stop reflux. After completion of the procedure, a drop of topical ofloxacin was administered. Thereafter, topical ofloxacin drops were prescribed for seven days.

IOP measurements were taken 30 min after injection using GAT and recorded using a specially designed data collection form. The safety of the procedure was defined as an IOP ≤20 mmHg (using GAT) 30 min after IVB injection. The IOP of patients who did not fulfill the safety criteria was re-measured after another 30 min.

Data was entered and analyzed using the Statistical Package for the Social Sciences software version 22 for Windows (IBM, Armonk, NY, USA). Frequency and percentage were computed for qualitative variables such as safety and sex. The mean±standard deviation (mean±SD) was calculated for quantitative variables such as age, duration of diabetes, and baseline IOP. Safety was stratified according to sex, age, duration of diabetes, and baseline IOP. A post-stratification chi-square test was applied, and statistical significance was set at p-value <0.05.

## Results

A total of 101 patients, comprising 61 (60.4%) male and 40 (39.6%) female, who fulfilled the study criteria were included in the study (Table 1). 

**Table 1:** Frequency and percentage of patients according to sex

Sex	Frequency	Percentage
Male	61	60.4%
Female	40	39.6%
Total	101	100%

The age of all participating patients was between 30 and 75 years with a mean±SD age of 55.66±6.37 years as shown in Table 2. The mean±SD duration of diabetes and baseline IOP were 7.73±2.94 years and 15.40±1.77 mmHg, respectively (Table 2).

**Table 2:** Mean±SD of patients according to age, duration of diabetes, and baseline IOP SD, standard deviation; IOP, intraocular pressure

Demographics		Mean±SD
1	Age (years)	55.66±6.37
2	Duration of diabetes (years)	7.73±2.94
3	Baseline IOP (mmHg)	15.40±1.77

The safety of the procedure, which was defined as IOP ≤20 mmHg 30 min after IVB injection, is shown in Table 3. Of the 101 patients, 91 (90.1%) had an IOP below the safety limit, while 10 (9.9%) had an IOP >20 mmHg (Table 3).

**Table 3:** Frequency and percentage of patients according to safety

Safety	Frequency	Percentage
Yes	91	90.1%
No	10	9.9%
Total	101	100%

The stratification of safety with respect to sex, age, duration of diabetes, and baseline IOP was performed, and the chi-square test was applied. There was no significant association between patient safety and sex, age, or duration of diabetes (Tables 4-6).

**Table 4:** Stratification of safety with respect to sex

Sex	Safety		p-value
	Yes	No	0.479
Male	56(91.8%)	5(8.2%)	
Female	35(87.5%)	5(12.5%)	
Total	91(90.1%)	10(9.9%)	

**Table 5:** Stratification of safety with respect to age

Age (years)	Safety		p-value
	Yes	No	0.134
30-50	17(100%)	0(0%)	
51-75	74(88.1%)	10(11.9%)	
Total	91(90.1%)	10(9.9%)	

**Table 6:** Stratification of safety with respect to duration of diabetes

Duration of Diabetes (years)	Safety		p-value
	Yes	No	0.098
1-5	20(100%)	0(0%)	
>5	71(87.7%)	10(12.3%)	
Total	91(90.1%)	10(9.9%)	

However, the chi-square test showed a significant relationship between safety and baseline IOP, with all of the subjects having >20 mmHg of IOP post-procedure (no safety group) had a baseline IOP of >15 mmHg as shown in Table 7.

**Table 7:** Stratification of safety with respect to baseline IOP IOP,  intraocular pressure

Baseline IOP (mmHg)	Safety		p-value
	Yes	No	<0.001
10-15	56(100%)	0(0%)	
>15	35(77.8%)	10(22.2%)	
Total	91(90.1%)	10(9.9%)	

## Discussion

Retinal ischemic diseases resulting in neovascularization and retinal exudation have been treated with "off-label" IVB injections since 2005 [12]. To date, no serious complication has been reported, which is only associated with IVB injection and is not observed with any other intravitreal injection.

We focused on the transient increase in IOP after IVB injection in patients with PDR, which could be a possible short-term complication of the procedure. In our study, it was shown that a 0.05 mL injection of IVB is safe to administer in patients with PDR with respect to transient IOP spikes. Thirty minutes after the injection, the majority (90.1%) of participants had an IOP ≤20 mmHg. However, the IOP measured in 10 patients (9.9%) at 30 min was between 20 and 25 mmHg. Upon rechecking IOP after another 30 min, it decreased to ≤20 mmHg in all patients. Another study conducted by Farhood QK, et al. on 52 diabetic patients (aged 28-75 years) reported similar results [10]. According to them, the frequencies of normal and increased IOP were 78.8% and 21.2%, respectively, 30 min after IVB injection for PDR.

In our study, before applying the chi-square test to check for the relationship between safety and other variables such as sex, age, duration of diabetes, and baseline IOP, all safety data collected were stratified. Patients were divided into two groups based on sex: male and female. Similarly, strata were created for age, duration of diabetes, and baseline IOP. Two groups were created for patients 30-50 years and 51-75 years. Patients with diabetes for 1-5 years and >5 years were grouped separately. Moreover, all patients with baseline IOP between 10 and 15 mmHg were placed in one group and those with baseline IOP >15 mmHg were placed in another group. There was no significant relationship between safety and sex, age, or duration of diabetes. However, a significant association was found between safety and baseline IOP. Patients with a baseline IOP between 10 and 15 mmHg were less likely to have an IOP >20 mmHg 30 min after IVB injection, whereas those with a baseline IOP >15 mmHg were at higher risk.

Transient spikes in IOP are common after IVB injections [10]. The literature also shows a transient increase in IOP with intravitreal injections of other anti-VEGF agents, which returns to <25 mmHg within 30 min to one hour without any pressure-lowering drugs [13,14]. The increase in IOP after intravitreal injection is greater in phakic eyes than in pseudophakic or aphakic eyes [15]. Our study also showed that the sudden increase in IOP was short-lived and returned to the normal range in all patients after one hour. All patients in this study were phakic, and those with any history of ocular surgery were excluded. Therefore, routine prophylaxis for IOP spikes is not mandatory, as the change in IOP is short-term and returns to normal without any early complications [16]. Based on these findings, it is also inappropriate to frequently check IOP after IVB injection in every patient because the chances of high IOP spikes are rare [10]. Late complications remain unidentified. However, in patients with advanced glaucomatous optic neuropathy, a careful approach should be adopted, and prophylactic measures such as anterior chamber paracentesis or anti-glaucoma medication should be considered after explaining this to the patient [17].

A study measuring immediate changes in IOP after a 0.1 mL injection of intravitreal triamcinolone acetonide (IVTA) reported that approximately 7% of participants had an IOP >25 mmHg measured at 30 min [18]. In our study, 9.9% of the participants had an IOP >20 mmHg after 30 min, but none crossed 25 mmHg. The reason for this is believed to be the administration of an extra volume (0.1 mL of IVTA ) compared to the half volume (0.5 mL of IVB) into the vitreous cavity. Another reason for this difference is the well-known side effect of steroids in the eyes, causing increased IOP through multiple mechanisms [19,20].

IOP changes after IVTA injection have been studied by many researchers [14,17-20]. We explored IOP fluctuations after IVB injection in a prospective manner with comparable sample sizes to check for the short-term effects of IOP on IVB injections [10,15]. We stratified the data according to sex, age, duration of diabetes, and baseline IOP and then analyzed the data, which adds to the strength of our study. However, this research has several limitations, such as consecutive sampling, no control group, and inability to quantify the reflux of the drug after injection owing to the transparent nature of the drug, similar to a previous study involving IVB injection [10]. However, drug reflux after IVTA injection has been measured by some scientists because of its milky-white color [18].

## Conclusions

Immediate IOP upsurge is a common and potential complication of intravitreal injections, including IVB injections. This study concluded that IVB injection is safe with regard to post-injection IOP fluctuation. It is a transient event without any serious short-term complications and the increase in IOP returns to the normal range in the majority of patients within 30 min after the procedure. Hence, the regular use of prophylactic anti-glaucoma medications in every patient is not advisable. However, there is a high chance of increased IOP 30 min after IVB injection in patients with a high baseline IOP. Therefore, it is advisable to evaluate IOP after IVB injection, especially in patients with a history of high-tension glaucoma. Prophylactic anterior chamber paracentesis or antiglaucoma drugs should be considered in such patients to ensure safety.
